# Genome data of four *Pythium insidiosum* strains from the phylogenetically-distinct clades I, II, and III

**DOI:** 10.1186/s13104-021-05610-y

**Published:** 2021-05-21

**Authors:** Theerapong Krajaejun, Weerayuth Kittichotirat, Preecha Patumcharoenpol, Thidarat Rujirawat, Tassanee Lohnoo, Wanta Yingyong

**Affiliations:** 1 Department of Pathology, Faculty of Medicine, Ramathibodi Hospital, Mahidol University, Bangkok, Thailand; 2 Systems Biology and Bioinformatics Research Group, Pilot Plant Development and Training Institute, King Mongkuts University of Technology Thonburi, Bangkhuntien, Bangkok, Thailand; 3 Interdisciplinary Graduate Program in Bioscience, Faculty of Science, Kasetsart University, Bangkok, Thailand; 4 Research Center, Faculty of Medicine, Ramathibodi Hospital, Mahidol University, Bangkok, Thailand

**Keywords:** *Pythium insidiosum*, Pythiosis, Genome sequence, Next-generation sequencing

## Abstract

**Objectives:**

We employed the Illumina NGS platform to sequence genomes of 4 different strains of the pathogenic oomycete *Pythium insidiosum*, the causative agent of pythiosis. These strains were isolated from humans in Thailand (n=3) and the United States (n=1), and phylogenetically classified into clade-I, -II, and -III. Our study augmented the completeness of the *P. insidiosum* genome database for exploration of the biology, evolution, and pathogenesis of the pathogen.

**Data description:**

One paired-end library (180-bp insert) was prepared from a gDNA sample of *P. insidiosum* strains ATCC200269 (clade-I), Pi19 (clade-II), MCC18 (clade-II), and SIMI4763 (clade-III) for whole-genome sequencing by Illumina HiSeq2000/HiSeq2500 NGS platform. A range of 28.459.4 million raw reads, accounted for 3.07.3Gb, were obtained and assembled into the genome sizes of 47.1Mb (15,153 contigs; 85% completeness; 19,329 open reading frames [ORFs]) for strain ATCC200269, 35.4Mb (14,576 contigs; 83% completeness; 13,895 ORFs) for strain Pi19, 34.5Mb (11,084 contigs; 84% completeness; 13,249 ORFs) for strain MCC18, and 47.1Mb (15,162 contigs; 85% completeness; 19,340 ORFs) for strain SIMI4763. The genome data can be downloaded from the NCBI/DDBJ databases under the accessions BCFN00000000.1 (ATCC200269), BCFS00000000.1 (Pi19), BCFT00000000.1 (MCC18), and BCFU00000000.1 (SIMI4763).

## Objective

Next-generation sequencing (NGS) is a sophisticated technology that facilitates multiple genome sequencing of different strains of the same microbial species, in a short duration, and at a low cost [1]. Obtained data promise extensive comparative genomic analyses to better understand the biology, evolution, and pathogenesis of a pathogen of interest. Besides, such data could serve as a comprehensive genetic resource for the identification of diagnostic and therapeutic microbial markers. Here, we employed the Illumina HiSeq2000/HiSeq2500 NGS platform to sequence the genomes of 4 different strains (i.e., ATCC200269, Pi19, MCC18, and SIMI4763) of *Pythium insidiosum*, a prominent pathogenic oomycete microorganism that infects humans and animals worldwide and causes an infectious condition with high mortality and morbidity, called pythiosis [2,4]. These strains were isolated from human patients with pythiosis from Thailand (n=3) and the United States (n=1), and have been phylogenetically classified into clade-I (n=1), clade-II (n=2), and clade-III (n=1), based on the ribosomal deoxyribonucleic acid (rDNA) sequence analysis [5]. So far, the draft genome sequences from 7 strains of *P. insidiosum* (including the synonym species *Pythium destruens*), isolated from humans, horses, and the environment in various countries, are available in the public databases [6,12]. This study contributed additional genomic data to augment the completeness of the public *P. insidiosum* genome database. Researchers around the world can use this genome data as a basis to explore the biology, evolution, and pathogenesis of *P. insidiosum*, which could provide knowledge that can be adapted for the development of preventive measures, reliable diagnostic assay, and effective therapeutic modality for pythiosis.

## Data description

The *P. insidiosum* strain ATCC20026*9* (phylogenetic clade-I) was isolated from a human patient in the United States, while the strains Pi19 (clade-II), MCC18 (clade-II), and SIMI4763 (clade-III) were isolated from human patients in Thailand. The identity (i.e., species) and genotype (i.e., clade) of each strain were confirmed by the rDNA sequence analysis [accession numbers: AB898108 (for strain ATCC200269), AB898113 (Pi19), AB971183 (MCC18), and AB971189 (SIMI4763)] [5]. These organisms were cultured in Sabouraud dextrose broth with shaking (50150 rounds per min) for one week at 37C. The resulting hyphal material of each strain was harvested and subjected to genomic deoxyribonucleic acid (gDNA) extraction, using an established method [13]. The identity of each strain was re-assessed by the rDNA sequence analysis, using the obtained gDNA [5]. One paired-end library with a 180-bp gap was prepared for each gDNA sample before proceeding to whole-genome sequencing by the Illumina HiSeq2000 (for strains Pi19 and MCC18) and HiSeq2500 (for strains ATCC200269 and SIMI4763) NGS platforms (Yourgene Bioscience, Taiwan), as previously described [6, 7, 10, 12]. In brief, the Qiagen CLC Genomics Workbench software trimmed raw reads to ensure a read length of at least 35 bases. Cutadapt 1.8.1 [14] removed the adaptor sequences from all reads. A total of 59,442,302 raw reads (average length: 122.2 bases) from the strain ATCC200269; 30,517,195 raw reads (average length: 92.5 bases) from the strain Pi19; 28,443,839 raw reads (average length: 94.7 bases) from the strain MCC18; and 28,531,434 raw reads (average length: 122.3 bases) from the strain SIMI4763 were obtained. Velvet 1.2.10 [15] assembled the raw reads of the strain ATCC200269 into 15,153 contigs [average length: 3111.1 (range: 300182,581); N50: 11,266; total bases: 47,142,494; %N: 0.7%; genome coverage: 154]; the strain Pi19 into 14,576 contigs [average length: 2426.8 (range: 300111,336); N50: 6208; total bases: 35,372,432; %N: 2.4%; genome coverage: 91]; the strain MCC18 into 11,084 contigs [average length: 3116.3 (range: 300150,908); N50: 8946; total bases: 34,541,218; %N: 2.3%; genome coverage: 87]; and the strain SIMI4763 into 15,162 contigs [average length: 3109.2 (range: 300182,337); N50: 11,187; total bases: 47,141,692; %N: 0.7%; genome coverage: 74]. BLAST search analyses of the assembled sequences of the strains ATCC200269, Pi19, MCC18 and SIMI4763, using the Core Eukaryotic Genes Mapping Approach (CEGMA) panel (containing 248 highly-conserved eukaryotic genes) [16] demonstrated 85%, 83%, 84%, and 85% genome completeness, respectively. MAKER2 pipeline [17] assigned 19,329; 13,895; 13,249 and 19,340 open reading frames (ORFs) in the genomes of the strains ATCC200269, Pi19, MCC18 and SIMI4763, respectively. All contig sequences have been deposited in the National Center for Biotechnology Information (NCBI) and DNA Data Bank of Japan (DDBJ) databases under the accessions BCFN00000000.1 (for strain ATCC20026*9*), BCFS00000000.1 (Pi19), BCFT00000000.1 (MCC18), and BCFU00000000.1 (SIMI4763) (Table 1).

**Table 1:** Overview of data files/data sets

Label	Name of data file/data set	File types (file extension)	Data repository and identifier (DOI or accession number)
Data file 1	*Pythium insidiosum* strain ATCC200269, whole genome shotgun sequencing project	FASTA	GenBank (https://identifiers.org/ncbi/insdc:BCFN00000000.1) [18]
Data file 2	*Pythium insidiosum* strain Pi19, whole genome shotgun sequencing project	FASTA	GenBank (https://identifiers.org/ncbi/insdc:BCFS00000000.1) [19]
Data file 3	*Pythium insidiosum* strain MCC18, whole genome shotgun sequencing project	FASTA	GenBank (https://identifiers.org/ncbi/insdc:BCFT00000000.1) [20]
Data file 4	*Pythium insidiosum* strain SIMI4763, whole genome shotgun sequencing project	FASTA	GenBank (https://identifiers.org/ncbi/insdc:BCFU00000000.1) [21]

In summary, the draft genomes of *P. insidiosum* strains ATCC200269 (genome size: 47.1Mb), Pi19 (35.4Mb), MCC18 (34.5Mb), and SIMI4763 (47.1Mb) isolated from human patients with pythiosis living in Thailand and the United States, have been generated and publicly available. The obtained genome data could be a useful dataset to enhance the exploration of the biology, evolution, and pathogenesis of *P. insidiosum*, which can lead to clinical applications for better management of patients with pythiosis.

## Limitations

We used the Illumina HiSeq2000/HiSeq2500 short-read NGS platform to sequence 4 genomes of *P. insidiosum* (strains ATCC200269, Pi19, MCC18, and SIMI4763). Users of the genome data should be aware that the sequencing-by-synthesis technique in the Illumina platforms constructs a library base on DNA amplification, which could result in sequence coverage biases and substitution errors. As seen in the genome data of these *P. insidiosum* strains, the total bases ranged from 3.0 to 7.3Gb, and the genome sequence coverages ranged from 74 to 154. Another limitation of the study is the number and type of the DNA library. The genome sequences of each *P. insidiosum* strain were obtained from only one paired-end library. As expected, all strains showed a less complete genome (8385% CEGMA-based genome completeness), a higher number of contigs (11,08415,162 contigs), and a smaller genome size (34.547.1Mb), when compared with the *P. insidiosum's* reference genome (92% completeness; 1192 contigs; 53.2-Mb size) generated from one paired-end and three mate-pair libraries [8].

## Data Availability

Please see Table 1 and references [18,21] for details and links to the data. The draft genome sequence of the *P. insidiosum* strain ATCC200269 comprising 15,153 contigs (accession numbers BCFN01000001-BCFN01015153), is available in GenBank here: https://identifiers.org/ncbi/insdc:BCFN00000000.1 [18]. The draft genome sequence of the *P. insidiosum* strain Pi19 comprising 14,576 contigs (accession numbers BCFS01000001-BCFS01014576), is available in GenBank here: https://identifiers.org/ncbi/insdc:BCFS00000000.1 [19]. The draft genome sequence of the *P. insidiosum* strain MCC18 comprising 11,084 contigs (accession numbers BCFT01000001-BCFT01011084), is available in GenBank here: https://identifiers.org/ncbi/insdc:BCFT00000000.1 [20]. The draft genome sequence of the *P. insidiosum* strain SIMI4763 comprising 15,162 contigs (accession numbers BCFU01000001-BCFU01015162), is available in GenBank here: https://identifiers.org/ncbi/insdc:BCFU00000000.1 [21].

## Abbreviations

CEGMA: Core Eukaryotic Genes Mapping Approach
DDBJ: DNA Data Bank of Japan
gDNA: Genomic deoxyribonucleic acid
NCBI: National Center for Biotechnology Information
NGS: Next-generation sequencing
ORF: Open reading frame
rDNA: Ribosomal deoxyribonucleic acid
